# The Role of Micronutrients to Support Immunity for COVID-19 Prevention

**DOI:** 10.1007/s43450-021-00179-w

**Published:** 2021-09-02

**Authors:** Mukhtar H. Ahmed, Arez Hassan, Judit Molnár

**Affiliations:** 1 Sisaf Nanotechnology Drug Delivery, Ulster University, Belfast, BT37 0QB UK; 2 School of Medicine, Queen’s University, Belfast, BT9 7BL UK; 3 Faculty of Agricultural and Food Sciences, Széchenyi István University, 9200 Mosonmagyaróvár, Hungary

**Keywords:** Minerals, Nutrition, Vitamins, SARS-CoV-2, Supplements

## Abstract

The World Health Organization declared the novel coronavirus, named as SARS-CoV-2, as a global pandemic in early 2020 after the disease spread to more than 180 countries leading to tens of thousands of cases and many deaths within a couple of months. Consequently, this paper aims to summarize the evidence for the relationships between nutrition and the boosting of the immune system in the fight against the disease caused by SARS-CoV-2. This review, in particular, assesses the impact of vitamin and mineral supplements on the body’s defence mechanisms against SARS-CoV-2. The results revealed that there is a strong relationship between the ingestion of biological ingredients like vitamins C–E, and minerals such as zinc, and a reduction in the effects of coronavirus infection. These can be received from either nutrition rich food sources or from vitamin supplements. Furthermore, these macromolecules might have roles to play in boosting the immune response, in the healing process and the recovery time. Hence, we recommend that eating healthy foods rich in vitamins C–E with zinc and flavonoids could boost the immune system and consequently protect the body from serious infections.

**Graphical Abstract:**

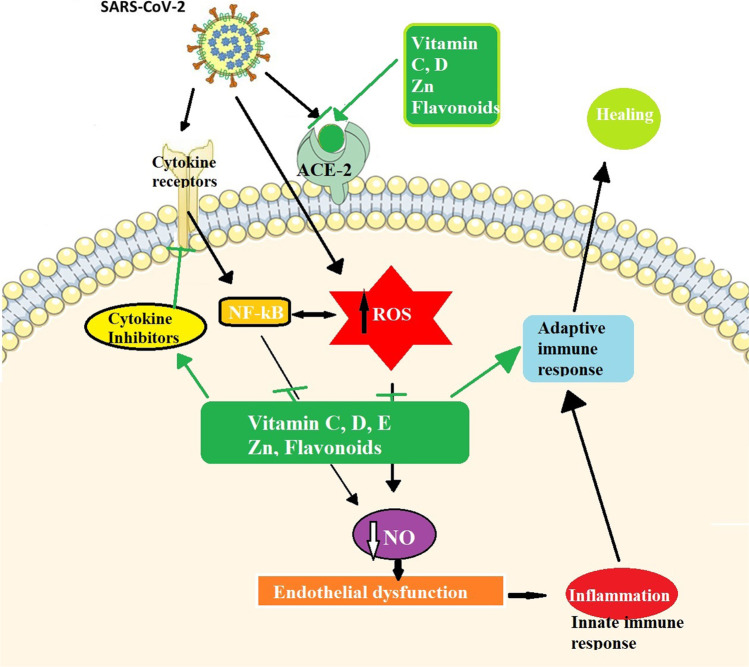

## Introduction

A new generation of coronavirus was detected in December 2019; it is similar to other generations of coronavirus such as MERS-CoV and SARS-CoV which are known to cause Middle East respiratory syndrome (MERS) and severe acute respiratory syndrome (SARS) in humans, respectively. The new coronavirus disease, which is known as COVID-19, is caused by the novel SARS-CoV-2 and can cause severe pneumonia and is dramatically more infective (Corman et al. 2019). The coronavirus is affecting almost all countries and territories around the world. At the time of writing, around 193 million cases of COVID-19 had been confirmed, and from which 4.2 million deaths had resulted.

Coronaviruses are enveloped viruses that possess a single-stranded, positive-sense RNA with a genome ranging from 26 to 32 Kb, the largest among all recognized RNA. Coronaviruses can be classified into four genera: alpha, beta, gamma and sigma coronavirus. Among these genera, the α-coronaviruses and β-coronaviruses mainly infect mammals and generally lead to respiratory disease in humans and gastritis in animals (Cui et al. 2019). Currently, there are many strains of coronaviruses that infect humans including HCoVHKU1, HCoV-NL63, HCoV-229E, SARS-CoV and MERS-CoV (Ghinai et al. 2020). The 3C-like protease (3CLpro), formally known as C30 endopeptidase, is the main protease found in coronaviruses. It cleaves the coronavirus polyprotein at eleven conserved sites (Rathnayake et al. 2020). The papain-like protease PLpro is an essential coronavirus enzyme that is required for processing viral polyproteins to generate a functional replicase complex and enable viral spread.

Since the start of the pandemic, several clinical trials have been used to determine the efficacy of certain existing anti-inflammatory and anti-viral drugs as alternative treatment for the disease (Bhatia 2021). Despite these attempts, there are still no drugs which promise to fully cure people from this new disease. Currently, several vaccines are being evaluated for their efficacy and safety and for determination of doses for COVID-19, and this requires considerable time for their validation (Ali et al. 2020; Forchette et al. 2021).

The use of alternative herbal remedies has gained prominence following their use in the management of COVID-19 especially in the west where attempts have been made to formulate anti-COVID-19 herbal products. This is a positive approach considering the ever-growing demand for natural, herbal alternatives for drugs and medicines (Chinsembu 2020; Komolafe et al. 2021). Thus, to avoid this disease or to get over the disease quickly, people have been redirecting their focus to natural foods to mimic the medical treatment, as well as to protect themselves from the virus. Following this, nutrition experts have been considering the types of food and diets which have a direct impact on reducing the infection and decreasing the mortality rate.

Healthy eating has been part of a healthy lifestyle and proper nutrition contributes greatly to the strengthening of the immune system. By increasing the body’s defences and maintaining it in an excellent condition, the coronavirus can be defeated more easily. Several studies have showed that the lack of quality and/or quantity of micro- and macronutrients (malnutrition) in the diet causes an increase in the morbidity and mortality (Jayawardena et al. 2020). Malnutrition resulted in an increased rate of viral and/or bacterial infections and delayed recovery in the living body. Recent studies have revealed that the importance of optimal nutritional status is necessary to protect against both bacterial and viral infections (Calder et al. 2020). The review focuses on the properties and immunological roles of vitamins C, D and E with zinc and flavonoids to support immunity for prevention of respiratory infections, acute respiratory distress syndrome (ARDS) and COVID-19.

## Search Strategy

Electronic searches were conducted on Google Scholar database Medline and PubMed. A further search was conducted on the World Health Organization’s COVID-19 research article database. The search items included coronavirus, SARS-CoV-2, supplements, nutrition and immune system. This review was conducted to analyse the recent literature to show the impact of micronutrition in preventing the coronavirus disease.

## Discussion

### Pathogeneses of COVID-19

Coronavirus are large family enveloped and positive strained RNA viruses that contain the largest known RNA genomes with a length of up to 31 kb. Coronaviruses pathogens cause various diseases in mammals (humans and animal) and birds, including the recently isolated severe acute respiratory syndrome coronavirus (SARS-CoV). The SARS-COV-2 S-protein is similar is that of SARS-COV, while the affinity of SARS-COV-2 for (ACE-2) receptor is approximately 20 times higher than that of the SARS S-protein (Ahmed and Hassan 2020). The results found that the immune cells such as monocytes and natural killer (NK) cells respond more strongly to viral infection. Adult respiratory distress syndrome (ARDS) starts to develop about after 7 days of the disease because of an explosive host immune response due to uncontrolled viral replication.

The SARS-CoV-2 infection has sequential phases, with the progression, from one phase to the next, causing the deterioration in the health of the patient. After the first 5 days of incubation in the body, the virus directly affects the respiratory system, the patient begins to show severe hypoxemia (a decrease in the partial pressure of oxygen in the blood) which later causes abnormally low oxygen content in the tissues and/or organs (Siddiqi and Mehra 2020). In the second phase of infection, granulocyte colony-stimulating factor (G-CSF), inflammatory cytokines and biomarkers like interleukin IL-2, IL-6, IL-7, tumour necrosis factor-α (TNF-α) macrophage inflammatory protein 1-α, D-dimer, C-reactive protein (CRP) and ferritin are markedly elevated in patients who are critically ill (Ahmed and Hassan 2020). In the third phase, patients, who are infected with COVID 19, are susceptible to developing shock, respiratory failure, cardiopulmonary collapse (Mehta et al. 2020), acute kidney injury (AKI) (McAdams et al. 2021), liver damage, gastrointestinal system tissue damage, blood clots and nervous system damage.

In general, following the viral multiplication, high numbers of reactive species (RS) can be produced, causing an imbalance in cellular redox homeostasis. Consequently, the imbalance of free radicals and antioxidants in the cell (oxidative stress) is caused by the viral infection, enhancing the production of reactive oxygen species (ROS) (Mrityunjaya et al. 2020; Camini et al. 2017). Therefore, changes in redox homeostasis in infected cells are one of the important events in the pathogenesis in all phases of the disease (Khomich et al. 2018). Besides the depression of the immune system, the lungs are the main organ which are most affected by coronavirus disease, as this virus mainly infects the upper and lower respiratory systems. In addition to that, some literature has revealed that acute kidney injury (AKI) is also associated with coronavirus disease (Kunutsor and Laukkanen 2020). A recent study showed that COVID-19 directly infects the heart and causes the myocardial injury, due to the virus binding to ACE-2 receptors in the cardiovascular system (Madjid et al. 2020)*.* Furthermore, the damage of other systems like the central nervous system, circulatory system, urogenital system and the digestive system have been recorded among severely ill patients with COVID-19 (Zhang et al. 2020). As well as that following the recovery from COVID-19, many patients, particularly the elderly and those with underlying medical conditions, are most likely to experience lingering COVID-19 symptoms like shortness of breath, muscle pain, headache fatigue and join pain. This condition is called long-COVID-19 syndrome (Ayoubkhani et al. 2021).

To reduce the above symptoms and shorten the recovery time of this disease and others, the nutrition experts have recommended eating healthy foods like those high in vitamins and minerals such as fresh vegetables and fruits, claiming that this could have significant impact in improving health and reducing the negative effects of the disease, during and post COVID-19 infection. This is because many micronutrients are able to protect or initiate the recovery of the damaged organs, caused by COVID-19, through boosting the immune system and fighting this virus during infection stage. As aforementioned, acute kidney injury (AKI) incidents occur in patients with COVID-19. This can be higher in some cases due to the higher prevalence of comorbid conditions like chronic kidney disease (CKD), diabetes and hypertension (McAdams et al. 2021). On the other hand, the results reported that anaemia is associated with AKI and its interstitial damage. This occurs as the injured kidney cannot make enough erythropoietin hormone (EPO); this hormone is required to stimulate haematopoiesis (formation of new red blood cells), and thus red blood cell count drops leading to anaemia. This disease causes additional problems to the patient include increasing the cardiac output, reduce oxygen utilization level, as well as affecting the responsiveness of the body’s immunity.

### Micronutrients in Immunity

The human immune system is essential for our survival. The immune system helps to protect our body against diseases caused by virus, bacteria and parasites by recognizing and responding to antigens, the substances that cause the immune system to produce antibodies against it. The immune system is divided up into two parts: the innate or (non-specific) immune system and the adaptive or (specific) immune system. They consist of different cells which work together properly to protect our bodies from foreign invaders by each being responsible for the destruction of different types of pathogens. The innate immunity response, particularly the type-I interferon (IFN), provides a general defence against harmful materials. This function is described as a first line of defence in the immune response. The adaptive immune system produces antibodies and uses them to fight certain foreign bodies that our body has previously encountered. The most essential components of the adaptive immune system are CD4 + T cells, CD8 + T cells and B cells (antibodies) (Nawsherwan et al. 2020).

In the case of COVID-19 attack, the innate immune system quickly recognizes the infection and is triggered within a couple of hours of infection. The innate immune response tries to directly inhibit the virus replication within infected cells and produces a local environment rich in IFN, in addition, to prime the adaptive immune response. After the priming effect, adaptive immune response takes 6–10 days to produce enough cells to control a viral infection (Sette and Crotty 2021).

### Vitamin C

**Scheme 1:**
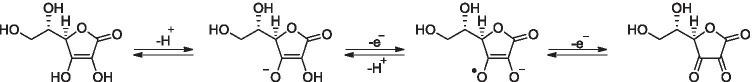
The structure of ascorbic acid and the main products of redox reactions. Ascorbic acid (AscH2); ascorbate anion (AscH¯); ascorbyl radical (Asc•^-^); dehydroascorbic acid (DHA)

The water-soluble micronutrient vitamin C, also known as ascorbic acid or ascorbate, has potential biological activity in the human body. It has the chemical formula C_6_H_8_O_6_. The chemical designation for ascorbic acid is 2-oxo-L-theo-hexono-4-lactone-2, 3-enediol. Ascorbic acid is a near planar five-member ring with two chiral centres that resolves into the four stereoisomers. As an electron donor, ascorbic acid acts as a cofactor for several mammalian enzymes that mediate a variety of essential biological functions, including wound healing and collagen synthesis (Messina et al. 2020). In adults, the requirement for vitamin C is 75–90 mg per day and a deficiency leads to impaired collagen synthesis, contributing to the more severe symptoms of scurvy. Vitamin C plays a vital role in the protection of cellular components like nuclease, protein, lipids and as well cell membranes from the oxidizing effects of free radical induced damage (Name et al. 2020). Furthermore, their activity is not only intracellular, but vitamin C is also responsible for immune activation following exposure to chemical and biological toxins. Another biochemical role of vitamin C is to act as an antioxidant (a reducing agent) by donating electrons to various enzymatic and non-enzymatic reactions. This activity converts vitamin C to an oxidized state, either as semi-dehydroascorbic acid or dehydroascorbic acid. These compounds can be restored to a reduced state by glutathione and NADPH-dependent enzymatic mechanisms (Scheme 1).

Several clinical studies have shown the ability of vitamin C to increase serum concentrations of iron and haemoglobin. Vitamin C is active in weak acidic and neutral (pH 4.5–7.4) conditions so vitamin C can act to increase the solubility of iron in the small intestine (duodenum) through chelation of ascorbic acid and iron to form iron chelate complex (Milman 2020). This is essential to ensure that iron-deficiency anaemia does not form, as this disease would be detrimental to those with COVID-19, a disease that already affects oxygen levels. Furthermore, ascorbic acid has the ability to protect against lipid peroxidation (LPO) by acting as a scavenger for reactive oxygen species and by causing a one-electron reduction of lipid hydroxyl radicals through the redox cycle of tocopherol (Traber and Stevens 2011).

In studies, assessing the link between vitamin C and various viral infections has shown positive results. In terms of the influenza virus, Kim and his group (Kim et al. 2013) demonstrated that vitamin C possesses a significant role in the *in vivo* antiviral immune response against the H3N2 influenza virus, via increased production of type I interferons (IFN-α/β), and their number increases within first couple of days after inoculation with a virus and, therefore, plays an essential role in the prohibition of viral pathogenesis. Furthermore, administration of vitamin C to patients with pneumonia induced by influenza virus (H1N1) showed a markedly reduced expression level of susceptibility genes, including interferon regulatory factor 3 (IRF3), mitochondrial antiviral signalling (MAVS), with an increase in nuclear factor kappa light chain enhancer of activated B cells (NF-κB) (Cai et al. 2015). Therefore, it might be logical to infer that the maintaining sufficient levels of vitamin C in the plasma either by the continuous uptake through the diet or supplement could effectively prevent *in vivo* pathogenesis of influenza virus at the initial stage of viral infection.

Vitamin C has shown to be effective against infections caused by other viruses, for instance, endothelial cells and human foreskin fibroblast were treated with ascorbic acid before cytomegalovirus (CMV) infection; there was a crucial decrease in the cellular viral load and the expression of viral antigen. This was explained by the immunomodulatory effects of ascorbic acid (Biancatelli et al. 2020a, b). Consequently, to reducing the level of infection, vitamin C has also been shown to be linked to a quicker recovery from fever, chills and chest pain as well (Ran et al. 2018). These results correlated with a meta-analysis study carried out on elective cardiac surgery patients, where (1–3) g/day of vitamin C was administered and resulted in a shorter the length of ICU stay, and the time mechanical ventilation was required (Hemilä and Chalker 2019).

Additionally, the earlier evidence of vitamin C to shorten the intensive care unit (ICU) stay of patients in critical care with conditions like respiratory failure and ARDS gives the opportunity to study the function of vitamin C in similar cases related to COVID-19 (Singh et al. 2020). Furthermore, in a systematic review of eight randomized controlled trials in 3135 children aged 3 months to 18 years, ascorbic acid administration showed to decrease the duration of upper respiratory tract infection by 1.6 days (Vorilhon et al. 2019). In a phase 1, randomized double-blind, placebo-controlled study, vitamin C infusion rapidly decreased the procalcitonin levels, and proinflammatory biomarkers C-creative protein (CPR) inhibited a rise in the level of thrombomodulin resulting in reduced vascular damage (Nawsherwan et al. 2020). A recent study demonstrated that administration of high-dose vitamin C, in combination with conventional treatment, such as anti-inflammatory (corticosteroids), antiviral and antibiotic drugs, was a safe and effective treatment for severe cases of patients with respiratory viral infection (Hoang et al. 2020). A further clinical study found that 4 g of vitamin C per day for 4 weeks in 594 severely ill surgical patients crucially reduced the incidence of acute lung injury and organ failure in comparison with patients receiving only a mechanical ventilation (Nathens et al. 2002). As a result, it is no surprising that the performance of high dose vitamin C treatment could significantly decrease the requirement for high doses of orthodox pharmaceutical drugs that may be immunosuppressive and cause disease complications. Therefore, vitamin C supplements promising to boost the immune system and promote resistance against COVID-19 infection. Hence, taking natural foods rich in vitamin C such as fresh vegetables and fruits like citrus fruits, strawberries, kiwi and cauliflower (Kalantar-Zadeh and Moore 2020) (Table 1), during COVID-19 pandemic, might be worthwhile. This is because it will help increase iron absorption, needed to prevent anaemia, and ensure adequate oxygen transport in the body, and help boost the immune system.

**Table 1:** Recommended daily amount and the sources of vitamins C, D and E with zinc and flavonoids

Macronutrients	RDA In adults	Overdosage	Nutrient sources	
			Plant	Animal
Vitamin C (ascorbic acid)	W:75 mg M: 90 mg (Institute of Medicine-US 2000)	˃ 2000 mg/day	Fruits: Blackcurrants, kale, citrus fruits (lime mandarin, orange), kiwi, Vegetables: Red pepper, tomato, broccoli, spinach, cabbage, strawberry	Low amount of vitamin C can be found in eggs, fish roe and raw liver
Vitamin D (1,25 cholecalciferol)	15 µg/day (Institute of Medicine-US 2011)	˃ 150 ng/ml in blood serum (Marcinowska-Suchowierska et al. 2018)	Edible mushrooms	Fish, meat, egg, and dairy like cheese
Vitamin E (α-tocopherol)	15 mg/day (Péter et al. 2015)	1000 mg/day (Owen and Dewald 2021)	Nuts and seeds: hazelnut, almonds, peanuts, sunflower, soya, vegetable oils, Fruits: mango, avocado	Low amount of vitamin E can be found in animal sources
Zinc	W:10 mg M: 15 mg (Gibson et al. 2016)	1.09 to 1.30 µg/ml (Agnew and Slesinger 2021)	Seeds and Nuts, Legumes: lentils, beans, and chickpeas Oa and whole grains	Oysters, red meat, and poultry
Flavonoids	250–400 mg (Peluso and Palmery 2015)	NA	Fruits: citrus fruits, berries, apples, cherries, grapes and soybeans Vegetables: onion and leafy vegetables Nuts, Tea and Wine	

Abbreviations: RDA is recommended daily amount, *W* women, *M* men

### Vitamin D

Vitamin D is a fat-soluble steroid hormone precursor that comes in two major forms: ergocalciferol which has the molecular formula, C_28_H_44_O (vitamin D_2_), and cholecalciferol which has the molecular formula, C_27_H_44_O (vitamin D_3_). Vitamin D_2_ is the active form of vitamin D_3_ and is produced in the kidney, while vitamin D_3_ is produced in the skin from conversion of 7-dehydrocholesterol by exposure of the sunlight. Ergocalciferol or vitamin D_2_ (**1)** and cholecalciferol or vitamin D_3_ (**2**) are equivalent for vitamin D production, as both forms appear to have similar efficacy in ameliorating rickets and reducing the incidence of falls in elderly patient. The metabolism and activation of vitamin D occur in the liver and kidney and magnesium as a co-factor. The liver converts cholecalciferol (**2**) to 25-hydroxy cholecalciferol [25(OH)Vit-D_3_] (**3**). The next step of the metabolism will occur in the kidney through the conversion of [25(OH) Vit-D_3_] to [1,25 dihydroxy Vitamin D_3_] (**4**) and [24,25 dihydroxy Vit-D_3_] (Stubbs et al. 2014). 
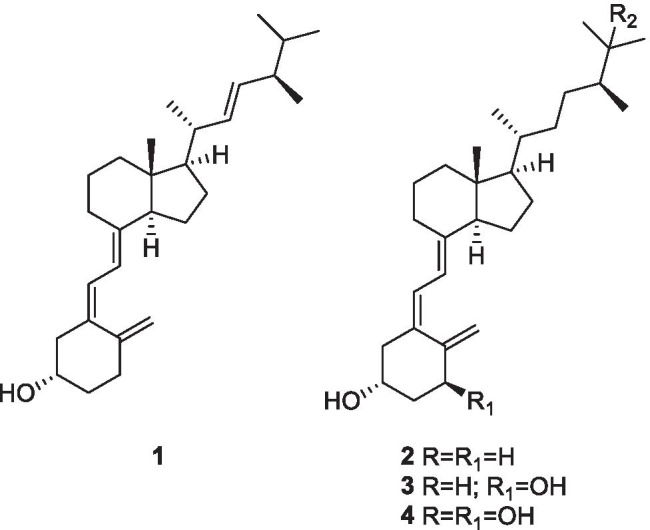


**Fig. 1:**
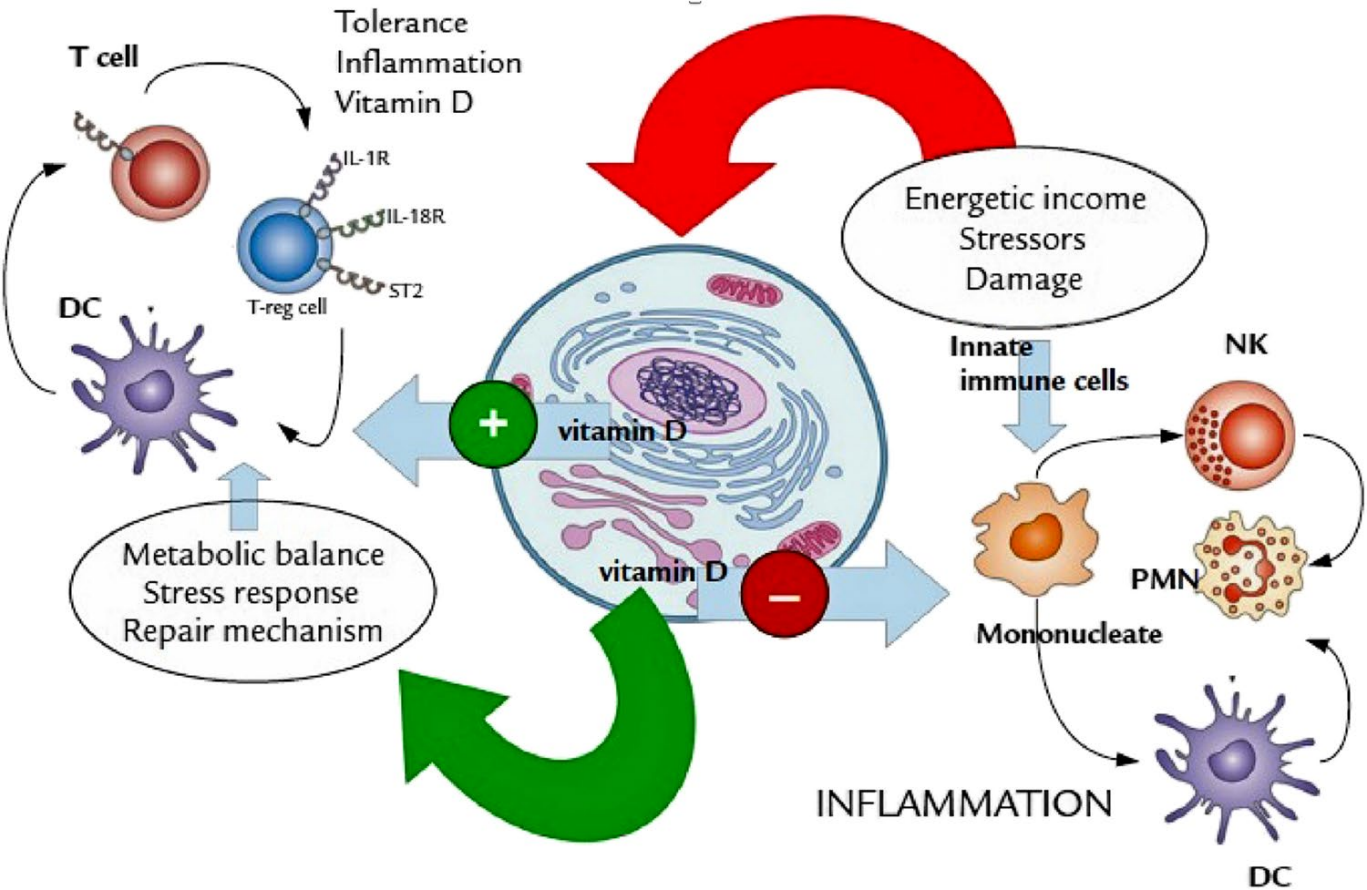
The anti-inflammatory and tolerogenic activity of Vitamin D. Reproduced from Chirumbolo et al. 2017

In addition to bone mineralization, vitamin D is also involved in cellular differentiation and regeneration of several organs. Vitamin D has a number of recently recognized roles within the immune system including endogenously regulating both the innate and humoral immune responses. Vitamin D, produced locally by monocytes, results in a shift in the immune status from proinflammatory to tolerogenic and regulates CD4 + T-cell responses by suppressing T helper (Th1) cell function and decreasing proinflammatory cytokines production, as illustrated in Fig. 1 (Bishop et al. 2021). In addition of helping dampen the immune system during a chronic reaction (anti-inflammatory potential), vitamin D also aids the innate immune cells to engulf and kill viruses or bacteria (Maggini et al. 2018). Furthermore, intake of vitamin D3 could enhances the amount of Treg cells (T cells) and reduce immunoglobulin-G (IgG) production (Rondanelli et al. 2018). Moreover, recent research studies have also found that 1,25 dihydroxy vitamin D_3_ (**4**) is considered as a transcriptional regulator of endothelial nitric oxide synthase, which leads enhanced endothelial nitric oxide (NO) production (Charoenngam and Holick 2020). Vitamin D deficiency in the body can be associated with various disorders, including skeletal deformities, cardiovascular disorders and metabolic syndrome (Uwitonze and Razzaque 2018). There is an inverse relationship between serum 25-hydroxy vitamin D3 levels and the upper respiratory tract viral infection. This might help conclude that the vitamin D3 supplements are associated with a lower risk of acute respiratory infections caused by COVID-19 (Shakoor et al. 2021). Vitamin D, similarly, to vitamin C, plays a key role in the formation and maintenance of epithelial barriers as lung tissue.

Pludowski and his group have suggested that vitamin D_3_ possess the ability to inhibit transcription of HIV RNA which is normally upregulated by TNF-α (Pludowski et al. 2018). Vitamin D_3_ has a key role in the regulation of both innate immune response (the first line of defence) and adaptive immune response, indicating that taking sufficient amount of vitamin D_3_ might protect patients with SARS-CoV-2 through blocking the activity of pro-inflammatory cytokines such as tumour-necrosis-factor (TNF-α), IL-6 production and interferon gamma (IFNγ) (Daneshkhah et al. 2020). On the other hand, vitamin D has been shown to exert an antioxidant effect by causing the upregulation of the nuclear factor erythroid 2-related factor 2 (NRF-2), a master inducer of antioxidant responses (Kim et al. 2020) which results in decreased ROS as well as the virus production (Mrityunjaya et al. 2020). Furthermore, in a randomized controlled trial, they found that a high vitamin D supplementation of (100,000) IU/month helps in decreasing the incidence of acute respiratory infections (Ginde et al. 2017). This result represents a good evidence to suggest that it may lead to a reduction in mortality in patients with COVID-19-associated ARDS, which is normally caused by increased expression of pro-inflammatory cytokines (Costela-Ruiz et al. 2020). Interestingly, a clinical study found that normal serum vitamin D_3_ may decrease SARS-CoV-2 infection severity, the time spent in intensive care units and mortality by around 50% (Kmietowicz 2020). A recent study revealed that vitamin D supplementation can lower the incidence, severity and risk of death from COVID-19 (Grant et al. 2020).

Therefore, sufficient vitamin D supplementation can enhance the immune system to prevent COVID-19 infection. The recommended daily allowance of vitamin D for adults is 15 µg per day (Institute of Medicine-US 2000). Thus, the consumption of foods rich in vitamin D and/or taking supplements, especially by those who live in northern countries who lack adequate sun light, are recommended to reduce the effects caused by the coronavirus infection. Foods that are particularly rich in vitamin D includemeat, egg, fish and dairy products such as cheese.

### Vitamin E

Vitamin E occurs in a variety of related fat-soluble isoforms, known as vitamers that include four tocopherols and four tocotrienols. Both the tocopherols and tocotrienols occur in α, β, γ and δ forms, as determined by the number and position of methyl groups on the chromanol ring. All eight of these vitamers feature a chromane double ring, with a hydroxyl group that can donate a hydrogen atom to reduce free radicals, and a hydrophobic side chain which allows for penetration into biological membranes (Niki and Abe 2019). Among those, only α-tocopherol (**5**) meets the human requirements for the active vitamin E isoform (Jovic et al. 2020). Of the many different forms of vitamin E, γ-tocopherol is the most common form found in the North American diet, but α-tocopherol is the most biologically active. Palm oil is a source of tocotrienols. 
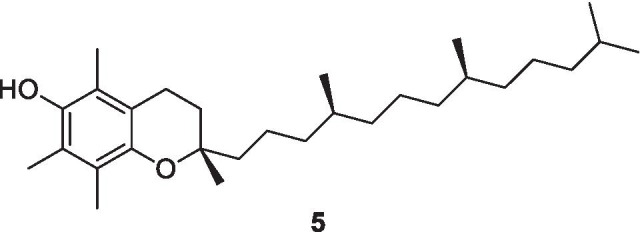


α-Tocopherol (**5**), a potent chain-breaking antioxidant, is an effective peroxyl radical scavenger; these properties play a significant role in protecting the nervous tissues and erythrocyte membranes as well (Traber and Atkinson 2007). Most of free radicals are produced from the metabolic reactions of unsaturated fats in the cell membranes, resulting in peroxidative decomposition. As it has been mentioned above, vitamin E supports the ability of vitamin C to protect against lipid peroxidation (LPO) (Halliwell and Gutteridge 2015).

Similar to flavonoids and ascorbic acid, α-tocopherol (**5**) is considered a strong antioxidant, which is capable of neutralizing reactive oxygen species (ROS) and can block acute neutrophil inflammation in the lungs (Sahin et al. 2020). Deficiency of α-tocopherol results in higher levels of lipid peroxidation; this has been confirmed in trials by the inverse relationship between α-tocopherol and plasma lipoperoxidase in acute respiratory disease syndrome (ARDS) patients. Results from randomized clinical studies have demonstrated that there is a significant relationship between α-tocopherol supplementation and a reduction in the incidences of upper respiratory tract infections in elderly nursing home residents (Meydani et al. 2004). α-Tocopherol acts via an antioxidant pathway to increase mitogenic lymphocyte response, enhance the number of T cells, increase natural killer (NK) cells activity (NK cells act to kill viral infected cells) (Sharrock 2019), and enhance IL-2 cytokine (also called T-cell growth factor, IL-2-related family that functions to stimulate T-cell growth) (Adolfsson et al. 2001). In addition, α-tocopherol supplementation showed the ability to enhance the resistance to respiratory infections (Kieliszek and Lipinski 2020). Although vitamin E appears to have some beneficial roles in immunity, there is limited clinical information on the effects of vitamin E in humans with COVID-19 infection, though patients are encouraged to have adequate intakes of all antioxidant nutrients. Vitamin E cannot be synthesized in the body so must be consumed through foods. Nuts, seeds and vegetable oils are the best source of vitamin E. The recommended daily dose of vitamin E is around 15 mg (Péter et al. 2015).

### Zinc

Zinc is considered a crucial trace mineral in the human body followed by iron. It is an essential cofactor for the synthesis and functioning of several proteins. It is a potent immunoregulatory agent, cytoprotectant and growth cofactor. In addition to that, it possesses strong anti-inflammatory, antiapoptotic and antioxidant stress agent activity (Zalewski et al. 2005), as it acts as a cofactor for around three hundred antioxidant enzymes (which convert the superoxide to hydrogen peroxide) in human body.

In terms of zinc’s role in antiviral immunity, zinc is involved in the innate immune response. Firstly, the antioxidant and anti-inflammatory activity of zinc makes it necessary for the barrier function of the mucosal epithelium. In addition, zinc controls the tight junction proteins, essential for ensuring the integrity of the mucosal membrane (Mossink 2020). A compromise in the integrity of the mucosa could exacerbate viral inflammation. This causes alveolar oedema, secondary to high weight protein and water exudation which may result in ARDs (Skalny et al. 2020). Furthermore, zinc regulates the proliferation, differentiation, maturation and functioning of T cells, B cells and eosinophils by modifying various signalling pathways, including T-cell receptor (TCR) signals, B cells receptor (BCR) and nuclear factor kappa B cells (NF-kB) signalling pathways, which plays a key role in controlling transcription of DNA, cytokine production and cell survival (Suzuki et al. 2021).

**Fig. 2:**
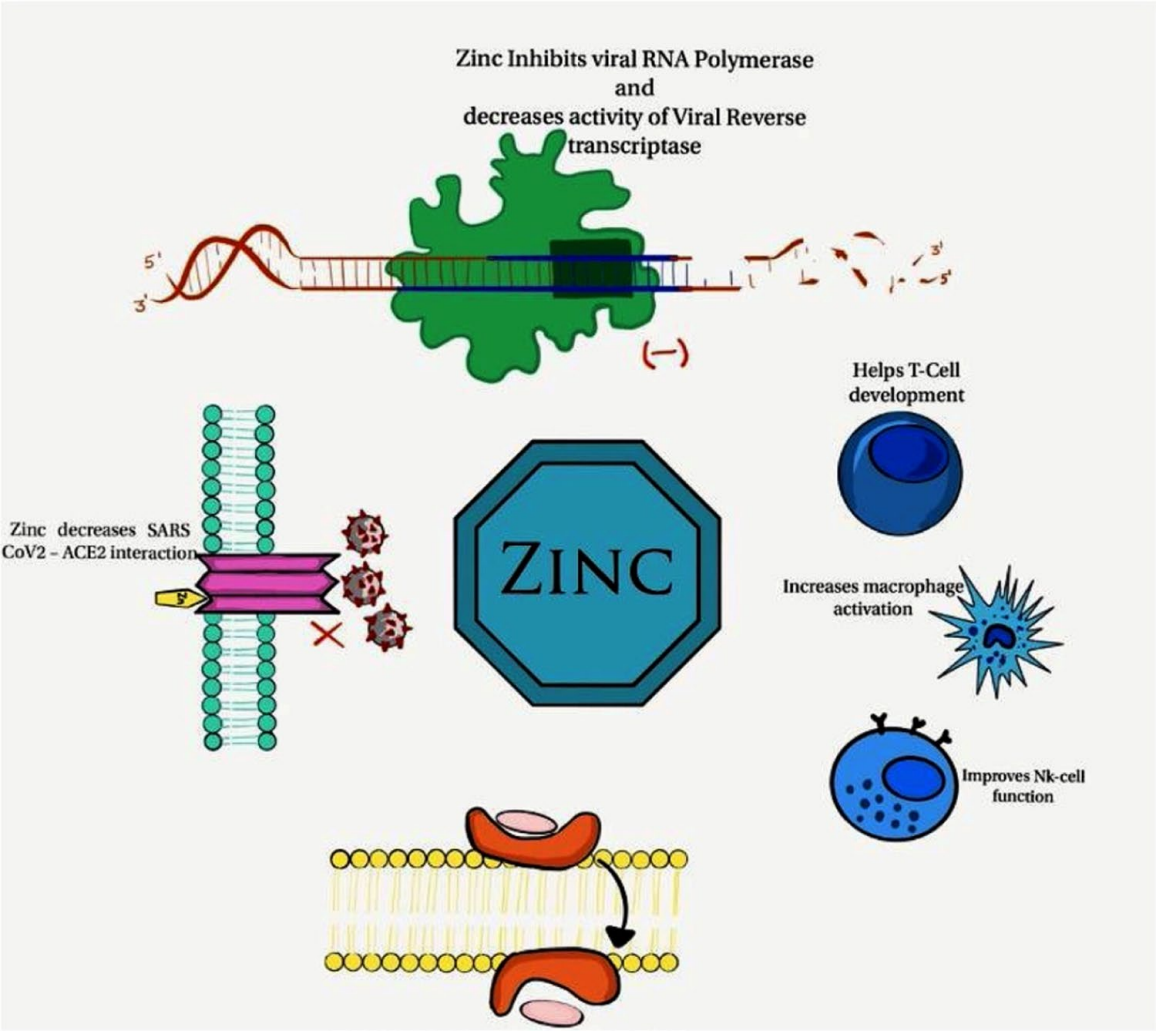
The various stages of viral replication cycles that are inhibited by zinc. Reproduced from Jothimani et al. 2020

Wessels et al. (2017) demonstrated that generation of cytokine and ROS depend on zinc availability in the cell. ROS play a significant role in physiological and pathophysiological signal transduction (Steven et al. 2018). Zn deficiency resulted in a serious alteration of lung epithelial cell function through up-regulation of TNFα, IFNγ and FasR signalling and cellular apoptosis (Skalny et al.^2020^). *In vitro* studies have shown that zinc therapy leads to increase in interferon-α secretion (IFN α) by leucocytes. This effect aids immune activation during a viral infection (Skalny et al. 2020). Studies demonstrated that oral supplementation of Zn decreases the incidence of acute respiratory infections by 35%. Furthermore, zinc reduces the duration of flu-like symptoms by 2 days and in addition improves the rate of recovery (Mrityunjaya et al. 2020). It modulates cytokine production, motivates proliferation of CD8 + T cells, develops T lymphocyte and prohibits free radical-induced injuries during the inflammatory response (Nawsherwan et al. 2020). Recent physiological studies have revealed that zinc is a strong inhibitor of HIV reverse transcriptase (RT) enzyme; this is also known as RNA-dependent DNA polymerase (Read et al. 2019). Recent investigations demonstrated that zinc is strongly involved in the modification and posttranslational process of proteins, when phosphorylation (addition of PO3¯ group on the surface of protein) is occurring, and ubiquitination which play a crucial role in many of the steps in innate immunity (Nchioua et al. 2020). The details of antiviral activities of zinc are illustrated in Fig. 2.

An *in vitro* study demonstrated that zinc inhibits the replication of coronavirus-19 in cell cultures (te Velthuis et al. 2010). In addition to that, recent study illustrated that zinc finger antiviral protein (ZAP), which efficiently targets CpG dinucleotides in viral RNA, significantly inhibits COVID-19 replication (Nchioua et al. 2020).

Mossink et al. suggested that increasing the level of zinc in the diet in all ages could play a significant role in controlling COVID-19 disease (Mossink, 2020).

Zinc deficiency has also been linked to an exacerbation of the reduction in smell and taste, which occurs in COVID-19 patients (Joachimiak and Joachimiak 2021). During an infection, an organism can mobilize zinc reserves for priority functions, such as those associated with the immune system. This leads to a decrease in zinc levels and possibly in the loss of zinc supply to other less essential functions, such as the maintenance of smell and taste. The recommended dietary allowance dose of Zn from various studies ranges from 10 mg up to 40 mg per day (Gibson et al. 2016). Oysters are considered the best source of zinc, following that, red meat, poultry, nuts, seed and legumes are also good sources of zinc (Table 1).

### Flavonoids

**Fig. 3:**
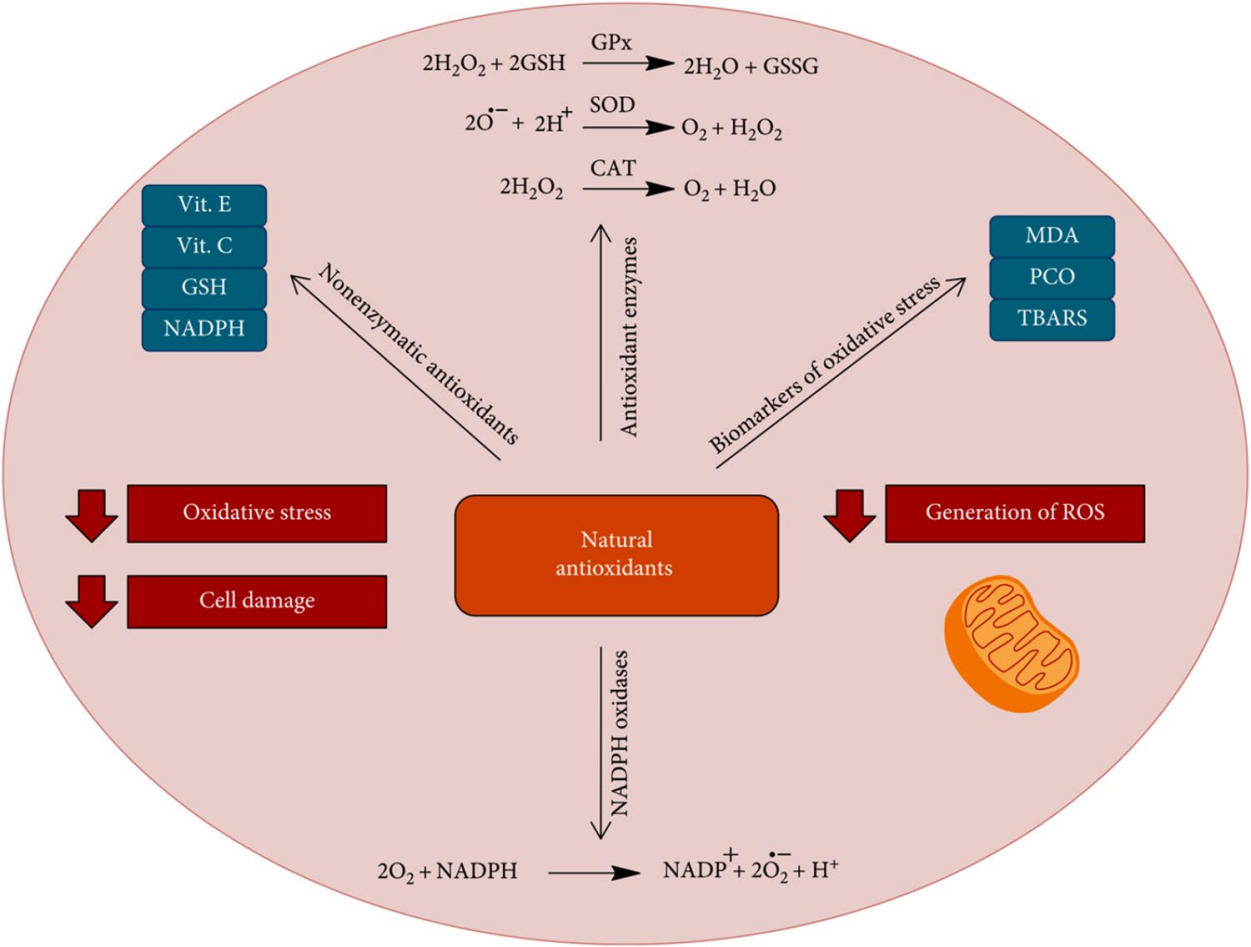
Antioxidant activity of active redox natural compounds as flavonoids (scavengers of ROS) by promoting an increase in the levels of antioxidant enzymes (SOD, CAT and GPx) and nonenzymatic antioxidants (vitamins C and E and GSH) and a reduction of oxidative stress, and cell damage. Abbreviations: superoxide dismutase (SOD), catalase (CAT), glutathione peroxidase (GSH), thiobarbituric acid reactive substance (TBARS), malondialdehyde (MDA), reactive oxygen species. Reproduced from Diniz et al. 2020

Flavonoids are a large family of polyphenolic compounds produced by plants. As a result of their lower redox potentials under the physiological conditions (− 30 mV to + 60 mV), they can oxidize automatically producing at least three or more oxygen free radicals (Eleonora et al. 2011). The action of free radicals leads to cellular membrane damage which causes cell death. Due to the capacity of the phenolic rings to promote the electron donation and hydrogen atom transfer to free radicals, acting as free radical scavengers, reducing agents and quenchers of single oxygen formation (Diniz et al. 2020), flavonoids possess potent physiological activities such as anticancer, antibacterial, anti-inflammatory and immunomodulatory abilities (Liskova et al. 2020). The antioxidant activity of flavonoids results in an increase in the levels of antioxidant enzymes which promotes a reduction of oxidative stress and cell damage as illustrated in Fig. 3.

Quercetin, 3,3',4′5,7-pentahydroxyflavone (**6**), is found in many plants and foods, such as red wine, onions, green tea, apples, berries, *Ginkgo biloba*, St. John’s wort, American elder and others. Buckwheat tea has a large amount of quercetin. Quercetin shows antioxidant activity at a concentration of 10 μmol/l in HepG2 cells, inhibiting oxidative stress promoted by H_2_O_2_; promotes an increase in superoxide dismutase (SOD), catalase (CAT), and glutathione peroxidase (GSH-PX); and reduces lipid peroxidation in rats with chronic prostatitis/chronic pelvic pain syndrome by anti-inflammatory and antioxidative activities through down-regulating nuclear factor kappa (NF-kB) and mitogen-activated protein kinase (MAPK) signalling pathways (Meng et al. 2018). Moreover, quercetin improves sepsis-induced acute lung injury in rats, by reducing lipid peroxidation and inflammation and increasing SOD and CAT levels. In addition, flavonoids showed significant reduction in interleukin (IL)-6 and IL-1β in lung tissue which are the proinflammatory cytokines (Rungsung et al. 2018). It has also been demonstrated that flavonoids are very strong nitric oxide (NO) producers (Van Acker et al. 1995). Nitric oxide is able to deactivate the coxsackievirus protease 3C enzyme, which is needed for the replication of coxsackievirus. This mechanism of inhibition of protease activity leads to a decrease in the viral life cycle (Saura et al. 1999). 
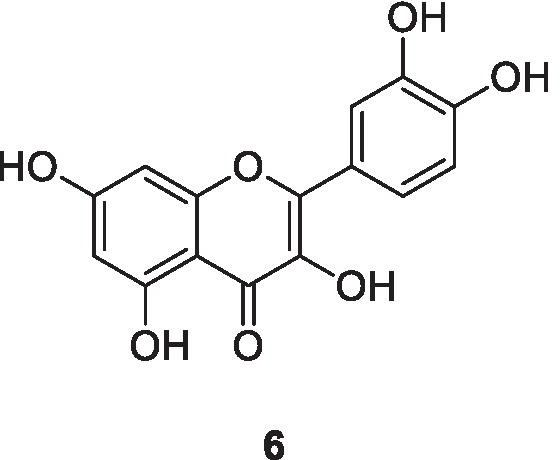


Antiviral properties of flavonoids have been demonstrated in numerous investigations. Castrillo and Carrasco (1987) reported that flavonoids able to bind to specific proteins during the transcription process of viral RNA. For example, 3-*O*-methylquercetin, 3',4',5,7-tetrahydroxy-3-methoxyflavone, inhibits selectively poliovirus RNA synthesis both in infected cells and in cell-free systems. Moreover, in a number of cases, flavonoids can bind to the viral capsid protein and modify the virus structure (Lalani and Poh 2020). Flavonoids can act at different stages of coronaviral infection, particularly at the molecular level to inhibit viral growth. Their antiviral activity could be attributed to its inhibitory effects on the enzymatic activity of target cells involved in coronavirus replication. The most important property of flavonoids is that they are able to display angiotensin converting enzyme (ACE2) inhibition activity. As explained above, ACE-2 is an effective receptor for coronavirus. It is found on the surface of the target cells and can strongly bind to the spike protein of coronavirus; this allows the virus to infect the epithelial target cells (Muchtaridi et al. 2020). A recent study demonstrated that flavonoids are able to inhibit both coronavirus protease enzymes 3C-like-protease (3CLpro) and papain-like cysteine protease (PLpro) which play an essential role in viral replication in host membranes (Russo et al. 2020). On the other hand, studies have observed that flavonoids contribute to the reduction of ROS accumulation decelerates the coronavirus-activated apoptotic signalling (Diniz et al. 2020; Godinho et al. 2021). In silico molecular docking studies have been performed to evaluate and validate the inhibitory effect of flavonoids based on the hydrogen-bonding distance between selected amino acid residues of the catalytic site from 3CLpro and PLpro SARS-CoV-2 proteases and flavonoids (Leal et al. 2021).

Finally, a study, assessing the link between ascorbic acid (vitamin C) and flavonoids, showed that synergy occurred as antiviral activity was enhanced. This is potentially due to the capability of vitamin C to recycle flavonoids and increase its activity in inhibiting viral protein and RAN synthesis (Vrijsen et al. 1987). Recently, based on the clinical trial, it is proposed that the oral administration of 250–500 mg quercetin (**6**), 500 mg vitamin C for high risk and mild symptomatic subjects twice a day for 7 days and up to 3 g vitamin C and 500 mg quercetin twice a day for 7 days in ARDS patients (assisted ventilation/intubation) improves the overall recovery in SARS-CoV-2 subjects (Biancatelli et al. 2020a, b). This may be due to the fact that the quercetin binds to either the COVID-19 spike protein at its host receptor region or the spike protein of the human angiotensin-converting enzyme 2 (ACE2) receptor interface, inhibiting the virus entry to cells and disrupting host–virus interactions indicating its therapeutic potential (Smith and Smith 2020).

The recommended consumption of flavonoids varies from 250 to 400 mg/day (Peluso and Palmery 2015). Foods, like tea, cherries, berries, apples and citrus fruits, are the main sources of flavonoids. As well as that, onion and green leafy vegetables are rich in dietary flavonoids (Table 1) (Waheed Janabi et al. 2020).

## Perspectives and Future Directions

Several clinical data have illustrated that there is a relationship between dietary supplements and COVID-19-associated coagulopathy, disrupted immune response and mortality, reduced platelet count and prolonged prothrombin time, suggesting there are benefits from supplementation.

Very little research has been conducted on, for example, the side effects of an overdosage of lipid soluble vitamins, namely vitamin D and vitamin E, and the usage of the correct dose of some menials to prevent the development of the conditions listed above, which occur secondary to COVID-19. The incorrect dose of treatment might cause harm instead of aiding recovery. To understand more about these points, further randomized clinical studies are needed, to investigate the clear potential impacts of these kinds of effects on COVID-19.

## Conclusions

In conclusion, this review has demonstrated that micronutrients act as an immune system booster; they can be a treatment option for those who choose not to take medications. The deficiency of micronutrients causes disorder in the immune system. It recommends that consuming foods rich in vitamins C, D and E and minerals like zinc could provide a better lifestyle that can enhance immunity to help fight the diseases caused by bacteria, viruses and parasites. Several studies have suggested flavonoids as potential inhibitors of COVID-19 transmission. It has been found that there are strong links among vitamins C, D and E, zinc and flavonoids in improving the immune system. This can lead to protection of individuals and populations against developing a severe disease which thus has an impact on the COVID-19 disease process. With these proven relationships, it would be beneficial for dietary recommendations to be put in place throughout this pandemic to reduce the prevalence of deficiencies of these chemicals. This will ensure that chances of severe disease are reduced and more pivotally chances of survival are boosted.
